# Bond Behavior and Critical Anchorage Length Prediction of Novel Negative Poisson’s Ratio Bars Embedded in Ultra-High-Performance Concrete

**DOI:** 10.3390/ma18133182

**Published:** 2025-07-04

**Authors:** Zhao Xu, Chang-Ze Xu, Xian-Liang Rong, Jun-Yan Wang, Xue-Yuan Ma

**Affiliations:** 1 College of Civil Engineering, Tongji University, Shanghai 200092, China; 2 Shandong Provincial Communications Planning and Design Institute Group Co., Ltd., Jinan 250101, China; 3 School of Materials Science and Engineering, Tongji University, Shanghai 201804, China; 4 Key Laboratory of Advanced Civil Engineering Materials of Ministry of Education, School of Materials Science and Engineering, Tongji University, Shanghai 201804, China

**Keywords:** negative Poisson’s ratio rebar, ultra-high-performance concrete, bond behavior, critical anchorage length, bond failure mode, eccentric pull-out tests

## Abstract

Negative Poisson’s ratio (NPR) reinforcement offers a novel solution to the usual trade-off between strength gains and ductility loss. Incorporating NPR into ultra-high-performance concrete (UHPC) effectively overcomes the ductility limitations of structural elements. However, the interfacial bonding between NPR reinforcement and UHPC is not sufficiently studied, especially its patterns and mechanisms, impeding the application of the materials. In this paper, the effects of nine design parameters (rebar type, prestrain, etc.) on the bond performance of NPR-UHPC through eccentric pull-out tests are investigated, and a quantitative discriminative indicator *K*_c_ for NPR-UHPC bond failure modes is established. The results showed that when *K*_c_ ≤ 4.3, 4.3 < *K*_c_ ≤ 5.64, and *K*_c_ ≥ 5.6, the NPR-UHPC specimens undergo splitting failure, splitting–pull-out failure, and pull-out failure, respectively. In terms of bonding with UHPC, the NPR bars outperform the HRB400 bars, and the HRB400 bars outperform the helical grooved (HG) bars. For the NPR bars, prestrain levels of 5.5%, 9.5%, and 22.0% decrease *τ*_u_ by 5.07%, 7.79%, and 17.01% and *s*_u_ by 7.00%, 15.88%, and 30.54%, respectively. Bond performance deteriorated with increasing rib spacing and decreasing rib height. Based on the test results, an artificial neural network (ANN) model is developed to accurately predict the critical embedded length *l*_cd_ and ultimate embedded length *l*_ud_ between NPR bars and UHPC. Moreover, the MAPE of the ANN model is only 53.9% of that of the regression model, while the RMSE is just 62.0%.

## 1. Introduction

Ultra-high-performance concrete (UHPC) possesses superior mechanical properties and crack control capabilities, exceptional durability, and good constructability [1,2,3,4]. As an excellent cementitious material, UHPC has received widespread attention and has been increasingly applied in engineering projects in recent years [5,6,7,8]. UHPC is particularly advantageous for structures requiring a high strength-to-weight ratio, such as bridges, or those needing superior corrosion resistance, such as marine structures [9,10,11]. Despite the durability, strength, and crack control of UHPC on its own, existing rebar-reinforced UHPC (R/UHPC) has shown undesirable behavior. According to existing studies, UHPC enters a softening phase due to the debonding and pull-out of steel fibers, which causes stress concentration in the reinforcement. This often leads to rebar fracture, and R/UHPC flexural components fail after the initiation of one or two localized cracks (e.g., Hasgul et al. [12], Shao et al. [11], Gu et al. [13], Wang et al. [14], and Guo [15]). The R/UHPC members failed to provide significant warning of failure, had poor ductility, and revealed drift ratios as low as 1.8% (e.g., Yoo et al. [16], Shao et al. [11]).

Significantly increasing the reinforcement ratio or reducing the fiber volume fraction (*V*_f_) can improve the ductility of R/UHPC components [15]. However, this may also lead to a series of adverse effects. Specifically, increasing the reinforcement ratio leads to increased costs, a surplus load-bearing capacity of R/UHPC components, rebar congestion, and heightened construction challenges. Decreasing the fiber volume fraction results in premature cracking of R/UHPC components and challenges in crack control [14,15]. Therefore, the performance of currently used rebar (such as normal-strength (NS) rebar, e.g., HRB400, and high-strength (HS) rebar, e.g., HRB635) appears to be mismatched with that of UHPC.

**Figure 1:**
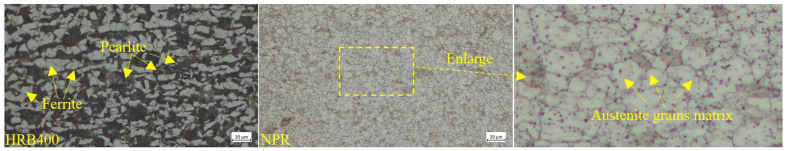
Metallographic structure of the NPR bars and HRB400 bars.

Recently, a new type of high-ductility, high-strength, and corrosion-resistant rebar referred to as meta steel with a negative Poisson’s ratio effect (named NPR rebar in this study) was developed [17,18]. NPR rebar overcomes the contradiction between the high strength and high ductility of conventional rebar [19,20]. The metallographic structures of the NPR and HRB400 bars are shown in Figure 1. Table 1 shows the measured chemical composition and content of the NPR and HRB rebars. Visibly, NPR rebars are austenitic materials, and the HRB400 rebars comprise pearlite and ferrite. Austenite exhibits good plasticity and high toughness, among other advantages. The Mn element content in the NPR reinforcement is 20.2%, while in the HRB400 reinforcement, it is only 1.44%. The Mn element is a stabilizer for austenite, enhancing its stability and allowing it to remain as retained austenite at room temperature. During the tensile process, the retained austenite in the NPR reinforcement undergoes continuous transformation-induced plasticity effects, and the twin density gradually increases, endowing NPR steel with superior uniform elongation [17,21]. The measured elongation before the fracture of the NPR reinforcement is 26.0%, which is significantly greater than that of HRB400 (18.3%). The C element content in NPR rebar is approximately twice that in HRB400 rebar. Research has shown that the C element is the most effective component for increasing rebar strength [17,18]. The measured yield strength *f*_y_ of the NPR bars is as high as 689 MPa, and the ultimate strength *f*_u_ is as high as 1139 MPa. The difference between the ultimate strength and yield strength *f*_u_ − *f*_y_ for NPR bars is 450 MPa, which is 3.33 times greater than that for HRB400 bars. This significant *f*_u_ − *f*_y_ difference provides a safety margin for structural components, effectively counteracting issues related to stress concentration and the tendency of reinforcements to fracture easily during the softening phase due to fiber debonding and pull-out, as well as the low ductility of R/UHPC components. Research by Gu et al. [13] has demonstrated that at the same reinforcement ratio *ρ*_ss_, the ultimate deformation of UHPC flexural components reinforced with NPR steel is 3.27 times (*ρ*_ss_ = 1.72%) and 1.71 times (*ρ*_ss_ = 2.58%) that of those reinforced with HRB400 steel.

From the above, it is evident that NPR bars are well matched with UHPC. Therefore, conducting research on NPR reinforcement and UHPC can promote the engineering application of UHPC and enhance the ductility of R/UHPC components. The bonding performance is the foundation for these two materials to work together and withstand loads together [22,23,24]. Bond performance plays a crucial role in the mechanical behavior of R/UHPC components under static and dynamic loads, such as in the cracking, deflection, and hysteresis performance of flexural members, especially in controlling crack width [25,26]. A low bond strength can result in wider cracks and may lead to sudden failure when the bond strength is insufficient, resulting in poor ductility. However, research on the bonding between NPR and UHPC is almost non-existent, which is detrimental to the engineering applications of UHPC.

In summary, in this study, an in-depth investigation of the bond performance between novel NPR bars and UHPC was conducted through eccentric pull-out tests, and quantitative discriminative indicators for bond failure modes were established. Based on the test results, a predictive model for the critical embedded length *l*_cd_ and ultimate embedded length *l*_ud_ between NPR and UHPC was developed using an artificial neural network (ANN). This research can provide supplemental data for standards and lay the foundation for the design and engineering application of R/UHPC structures.

## 2. Bonding Mechanism

**Figure 2:**
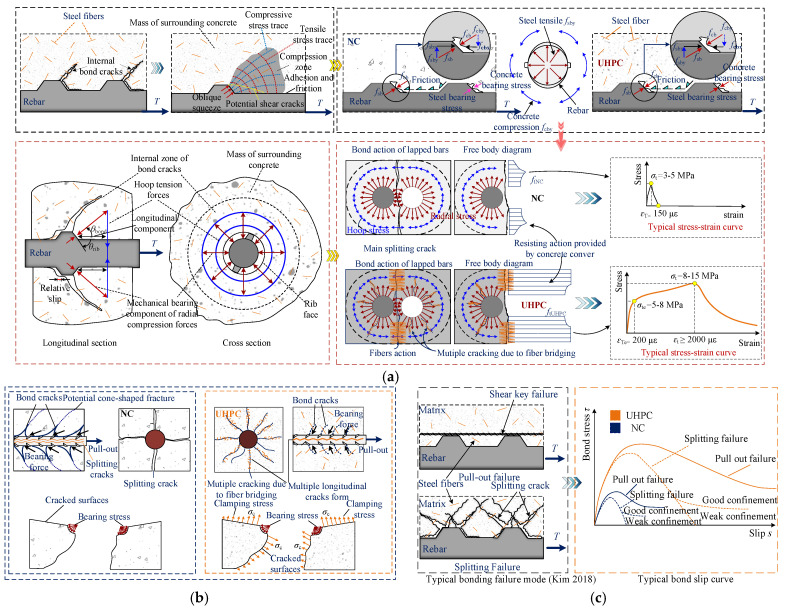
Bonding mechanism and failure modes. (**a**) Bonding mechanism [3,28,29]; (**b**) Cracking [27]; (**c**) Typical failure modes and *τ*-*s* curves [28,29].

The differences in the bonding mechanisms between UHPC and NC mainly lie in the different matrices and the crack-arresting effect of the steel fibers, as shown in Figure 2a,b. The packing density of UHPC significantly increases the tensile strength, enhances chemical adhesion and interlocking, strengthens the concrete interlock between ribs, and delays the propagation of splitting cracks. After the formation of internal oblique and radial cracks, with increasing tensile load, the radial compressive stress is redistributed to the matrix due to the presence of steel fibers [3], as shown in Figure 2a. At this stage, multiple microcracks form, and the strain-hardening properties of the UHPC materials control the extension of these microcracks, resulting in minimal or no decrease in the bond stress [27]. As the tensile load further increases, the fibers are gradually pulled out, and splitting cracks gradually develop along the direction of the rebar, which generally corresponds to the peak bond stress state. Thereafter, fibers either effectively bridge cracks, leading to matrix crushing around ribs and ductile pull-out failure, or permit progressive crack opening and brittle splitting failure as shown in Figure 2c.

## 3. Experimental Program

**Figure 3:**
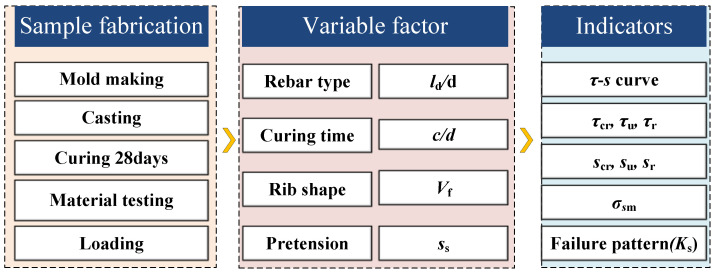
Experimental program.

The sequence of the experimental process is shown in Figure 3.

### 3.1. Rebar Material

**Figure 4:**
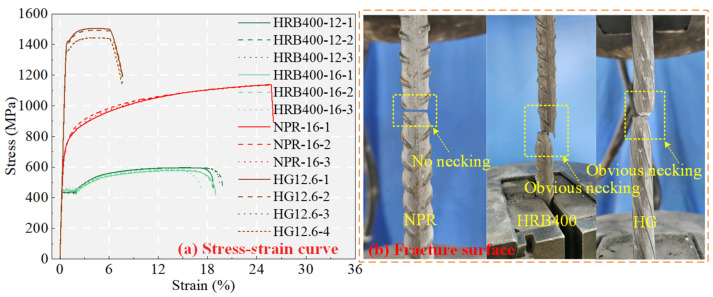
Rebar testing [31].

The shape and mechanical properties of the rebars significantly affect bonding performance. The shapes of the three rebars explored in this paper are shown in Figure 4. The NPR and HRB400 rebars share similar geometrical shapes. However, the helical grooved (HG) bars feature a continuous spiral shape without transverse ribs, influencing their bonding mechanisms with UHPC. For a detailed discussion about HG bars, see the study by Li et al. [30]. The measured mechanical properties of the rebar are shown in Figure 4, and the corresponding characteristic parameters are listed in Table 2.

Notably, the NPR bars exhibit no necking in the fracture state, and their fracture surface morphology markedly differs from that of the HRB400 and HG bars. This is mainly due to the twinning effect [17]. The stress of NPR bars experiences a slow increase stage before fracture. Their strength-to-yield ratio of 1.65 substantially surpasses the standard limit of 1.25 specified by GB/T 228.1-2010 [32] and ASTM A706 [33]. The difference between the ultimate strength and yield strength *f*_u_ − *f*_y_ for the NPR bars was 3.33 times that for the HRB400 bars and 5.29 times that for the HG bars. The elongation before fracture for the NPR bars is 26.0%, which is substantially greater than the 18.3% for the HRB400 bars and 7.6% for the HG bars, surpassing the 15% threshold mandated by GB/T 228.1-2010. Under tensile loading, the energy dissipation of NPR bars is approximately 2.8 times that of HRB400 and 3.0 times that of HG. In summary, using NPR bars instead of traditional mild steel as engineering structural steel has great advantages.

### 3.2. UHPC Material

The test involved four different fiber volumes *V*_f_ of UHPC, i.e., *V*_f_ = 0%, 0.5%, 1.2%, and 2.2%. The mix proportions of the UHPC and the parameters of the steel fibers are listed in Table 3.

**Figure 5:**
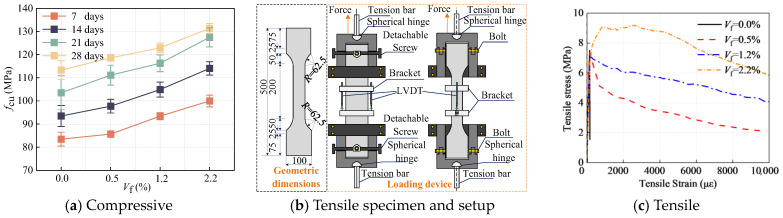
Compressive and tensile results [31,34].

The compressive strengths for these various *V*_f_ UHPC mixtures are depicted in Figure 5a. Concurrently, the tensile stress‒strain curves are presented in Figure 5c. These data indicate that the UHPC with *V*_f_ = 0.0% experiences a nearly vertical drop in tensile stress immediately following initial cracking. Similarly, UHPC with *V*_f_ = 0.5% and 1.2% exhibited significant strain-softening characteristics, especially for UHPC with *V*_f_ = 0.5%, for which the tensile strength suddenly decreased by 32% after initial cracking. The UHPC material with *V*_f_ = 1.2% did not show a sudden decrease in tensile strength after initial cracking but decreased slowly with increasing tensile strain. However, the UHPC with *V*_f_ = 2.2% exhibited typical strain-hardening behavior with an ultimate tensile strength of 9.18 MPa and an ultimate tensile strain of 2527.99 με.

### 3.3. Eccentric Pull-Out Design

**Figure 6:**
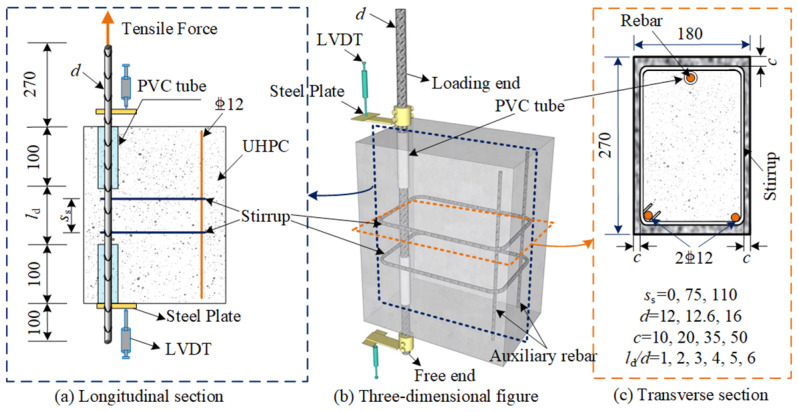
Specimen design (units: mm) [31].

To prevent the occurrence of lateral compressive stresses within the UHPC in compressed areas, the cracking of the UHPC was restricted, and the true bond strength was overestimated. Furthermore, to maintain the same cross-sectional reinforcement arrangement as in the actual structural components, eccentric pull-out tests are used. Figure 6 presents the specimen design.

In the specimen, a nonbonded zone of 100 mm in length (>6*d*) is established at the loading end using a polyvinyl chloride (PVC) tube to minimize the impact of the end restraint effects. Moreover, to prevent splitting failures at the ends of the embedment length, another 100 mm nonbonded zone is arranged at the free end. The cross-sectional dimensions and reinforcement of the eccentric pull-out specimen mirror those of the reinforced UHPC beam components. Further investigations into NPR-reinforced UHPC beams are detailed in Long’s research [34].

### 3.4. Test Process and Test Setup

**Figure 7:**
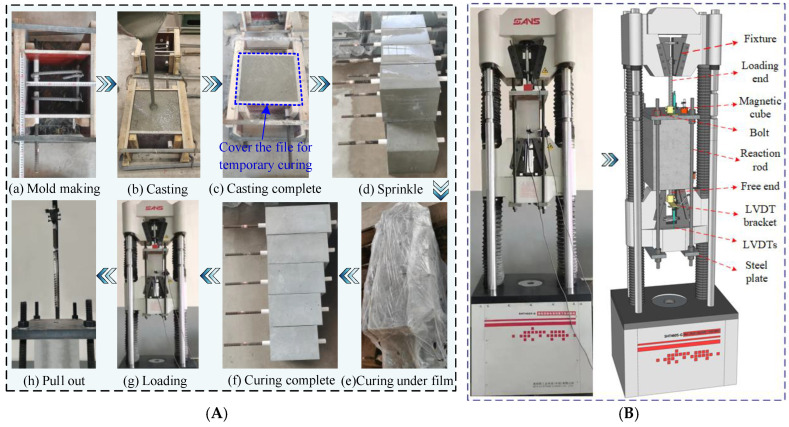
Test process and test setup details. (**A**) Test process [31]; (**B**) Test setup.

The test process is shown in Figure 7A. In order to avoid interference in determining the influence of design parameters on bond performance due to differences caused by the casting direction of steel fibers, all the specimens were cast parallel to the direction of the embedment rebar. This ensures that the measured bond strengths represent a conservative estimate. Shao’s [1] research demonstrated that UHPC cast perpendicular to the direction of rebars exhibits enhanced fiber bridging capabilities across the crack plane, which can increase the bond strength by 9–26%.

Figure 7B presents a schematic of the test setup. Two linear variable differential transformers (LVDTs) were arranged at the loading end and the free end. A displacement-controlled loading protocol was adopted for the test. For specimens with HRB400 and NPR rebars, the loading rate was set at 0.6 mm/min. For specimens with HG bars, the loading rate was set to 0.9 mm/min due to their large slip, which was usually greater than 45 mm. Notably, the displacement measured by the LVDT at the loading end includes the plastic deformation of the rebars, which overestimates the actual slip, potentially exceeding the true slip amount by more than double. Therefore, the slip reported in this study is based on the displacement measured by the LVDT at the free end.

### 3.5. Test Matrix

Table 4 presents the test matrix. Here, *d* is the rebar diameter, *l*_d_ is the anchorage length, *c* is the UHPC cover depth, and *s*_s_ is the hoop spacing. A bond length of 3*d* was selected as the control group because shorter bond lengths (less than 3*d*) exhibit high data scatter due to containing an insufficient number of ribs [35], while at greater bond lengths, the reinforcement within the UHPC would reach its yield state, thereby compromising its representativeness.

## 4. Test Results

### 4.1. Crack Pattern and Failure Mode

**Figure 8:**
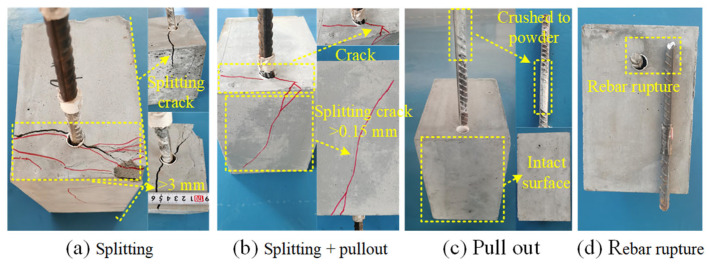
Failure modes.

When the circumferential and axial stresses exceed the tensile strength of UHPC, radial and internal diagonal cracks form. Due to the dense internal structure, low porosity and fiber reinforcement of UHPC, its bonding strength is significantly better than that of NC, which is approximately 4–5 times greater than that of NC [10]. Therefore, the UHPC matrix between the ribs of most specimens in this study is completely sheared and crushed into powder (see Figure 8c), resulting in pull-out failure (see Figure 8c). In view of the restraining effect of *c* and *s*_s_ and the fiber bridging effect, the radial crack reaches the surface of the specimen to form a small splitting crack (with a measured width < 0.3 mm), which ultimately causes splitting–pull-out failure, as shown in Figure 8b. In contrast, the splitting crack extends to the surface and causes the peripheral UHPC to disintegrate, resulting in splitting failure, as shown in Figure 8a. Splitting failures were observed in specimens K and Y, both without a stirrup (*s*_s_ = 0). The specimens without fibers (such as H) did not show the bursting failure pattern (UHPC split into multiple pieces and flew to all sides) described in Chang’s [20] study, which was mainly due to the effect of hoop restraint. Additionally, due to the lower strength of the HRB400 rebars, a failure mode of rebar fracture occurred in the experiment, as shown in Figure 8d. Figure 8 illustrates the typical failure modes, and Table 5 summarizes the failure modes of the specimens.

For specimens E and V with a cover thickness of 10 mm (*c*/*d* = 0.63), designed splitting failure did not occur. However, under the same *d*, *l*_d_, and *V*_f_ conditions, the center pull-out specimen (no hoops) with *c* = 12 mm (*c*/*d* = 0.75) experienced splitting failure [36]. Moreover, Sun et al. [8] suggested that the minimum value of UHPC cover *c* should be the larger value between 20 mm and *d*. Deng et al. [36] suggested that the minimum value of *c* should be the larger value between 10 mm and *d* for UHPC plate components and that the larger value should be between 15 mm and *d* for the UHPC beam and column components. Neither Sun nor Deng’s recommendations accounted for the impact of stirrup confinement [8,36]. Integrating these findings, our study concludes that a minimum UHPC cover of *c*/*d* > 1 is necessary to avoid splitting failure in non-hoop specimens. And a minimum UHPC cover *c*/*d* ≥ 0.63 is sufficient to prevent splitting failures in hoop confinement (*ρ*_ss_ ≥ 1.14) specimens.

**Figure 9:**
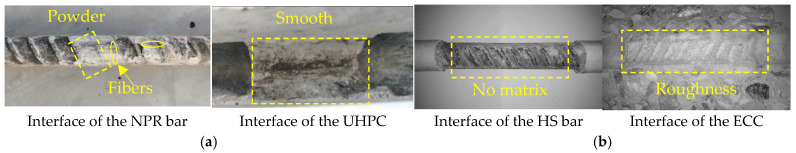
Contact interface of the rebar and matrix. (**a**) Interface of UHPC-NPR; (**b**) Interface of ECC-HS [37].

The UHPC specimens were cut after the test so that the contact interface between the matrix and the NPR bar could be observed. Figure 9a shows that the UHPC between the ribs was crushed into powder and smoothed, and the UHPC key between the ribs was sheared off. This indicates that the NPR-UHPC bond has fully developed and that bond failure is positively correlated with the shear strength of the matrix. Moreover, Deng et al. [37] examined engineered cementitious composite (ECC) specimens and noted that the concrete keys between the ribs remained intact, with no broken matrix observed, as shown in Figure 9b. This phenomenon suggests that the failure of the ECC specimens results from the insufficient tensile strength of the matrix [37].

### 4.2. Quantitative Discrimination of the Bond Failure Modes of NPR-UHPC

**Figure 10:**
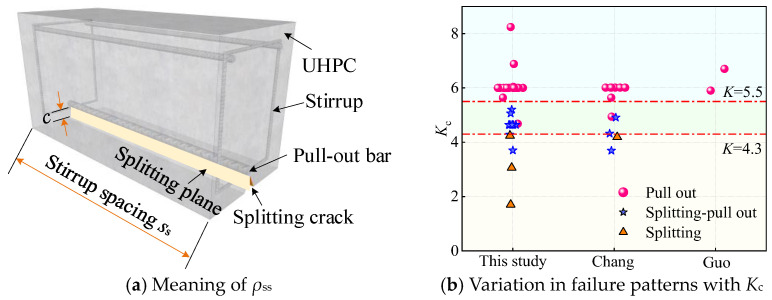
The meaning of *ρ*_ss_ and the variation in failure patterns with *K*_c_ [15,20].

To determine the bond failure mode of NPR rebar-reinforced UHPC specimens, the overall constraint parameter *K*_c_ is used as the discrimination index to characterize the failure type. *K*_c_ reflects the parameter that characterizes the overall constraint capability [38]. For UHPC specimens, *K*_c_ can be considered as a combined constraint parameter determined by the hoop, UHPC cover, and steel fibers, and its corresponding calculation equation is as follows:

(1) $$K_{c}=\lambda_{k}\rho_{\mathrm{ss}}+\frac{c}{d}+\lambda_{\mathrm{sf}}=\lambda_{k}\frac{A_{\mathrm{ss}}}{bs_{s}}+\frac{c}{d}+V_{f}\frac{l_{f}}{d_{f}}$$

where *λ*_k_ represents the conversion coefficient; *ρ*_ss_ is the area ratio of the stirrup area; *λ*_sf_ is the fiber characteristic parameter, *λ*_sf_ = *V*_f_(*l*_f_/*d*_f_); *l*_f_/*d*_f_ is the aspect ratio of the fibers; *l*_f_ and *d*_f_ are the fiber length and diameter, respectively; *b* is the specimen cross-sectional width; when there is no hoop, *λ*_k_*A*_ss_/*bs*_s_ = 0; and *A*_ss_ is the area of the hoop through the splitting plane, as shown in Figure 10a.

Figure 10b shows the variation in the failure modes of each specimen with *K*_c_. As shown in Figure 10b, with increasing *K*_c_, the failure mode of the specimen changes from splitting to splitting–pull-out and then from splitting–pull-out to pull-out. The corresponding critical values are 4.3 and 5.6 determined in this study for the NPR-reinforced UHPC specimens, respectively. In other words, when *K*_c_ ≤ 4.3, the NPR-reinforced UHPC specimens undergo splitting failure. When 4.3 < *K*_c_ < 5.6, they experience splitting–pull-out failure. When *K*_c_ ≥ 5.6, they exhibit pull-out failure.

### 4.3. Measured τ-s Curves

**Figure 11:**
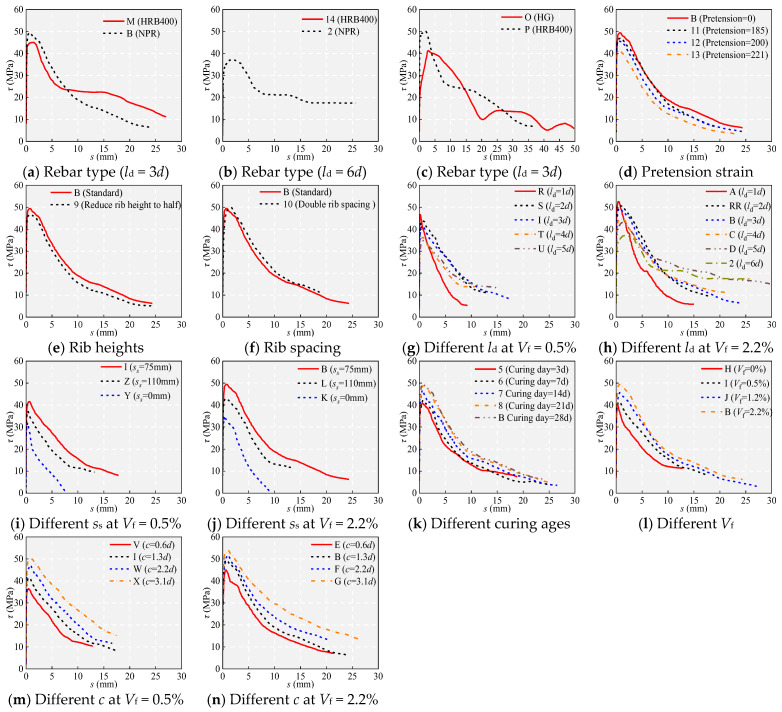
Measured curves.

**Figure 12:**
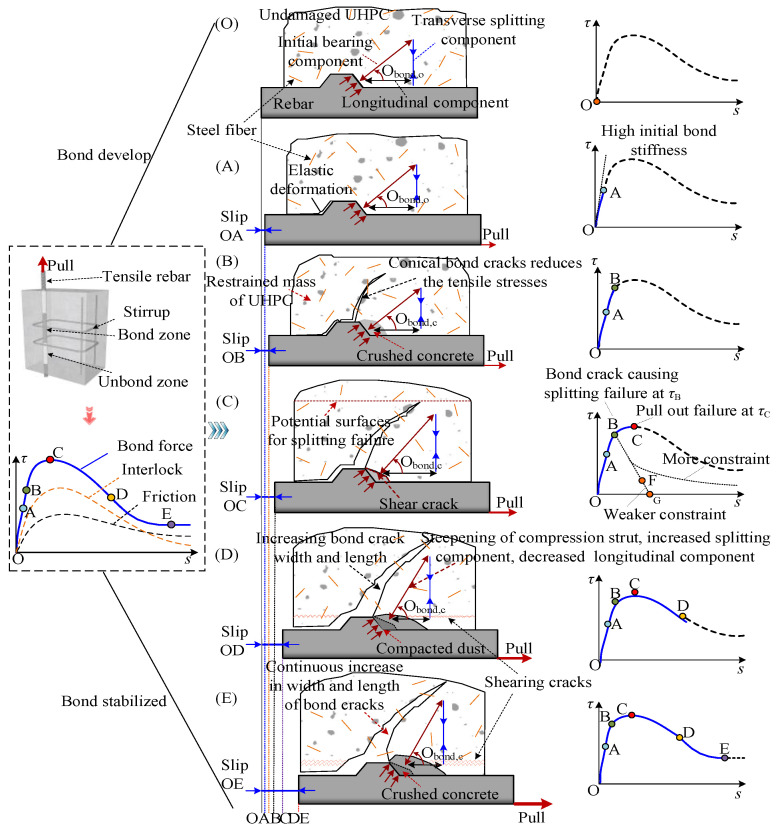
Ideal schematic of the bond response [28].

Figure 11 shows the measured *τ*-*s* curves. Figure 11 demonstrates that despite variations in parameters significantly influencing the *τ*-*s* curves, the overall stages of the *τ*-*s* curves for each specimen remain consistent. Figure 12 depicts an ideal schematic relationship extracted from the test results, which is believed to be suitable for reproducing the *τ*-*s* developing process. The movement of the rib is shown in Figure 12. The *τ*-*s* developing process can be categorized into five distinct phases.

(1) Elastic phase OA (<0.68 *τ*_u_)

In the initial loading phase, chemical adhesion is the predominant mechanism resisting the slippage of the rebars. As the tensile load increases, the loading end first breaks through the limitation of chemical adhesive force, and slip occurs, and the slip penetrates from the loading end to the free end. At this stage, the free end remains securely bonded, with the bonding forces predominantly composed of chemical adhesion and static friction. Due to the weak interlocking effect in the initial stage, the shear stress and strain along the surface of the rebars are in the elastic stage [30], and the matrix is basically not damaged. The weak slip at the loading end is mainly caused by the elastic deformation of the matrix (see Figure 12), so the slip is small. The bond stress‒slip relationship during this initial phase is considered to be in the elastic stage. Microcrack initiation occurs when the tensile stress applied to the rib-front matrix reaches the elastic limit tensile strength of UHPC (approximately 0.4–0.6 *τ*_u_) [24], and the bond stress‒slip relationship enters Stage II.

(2) Initial crack propagation phase AB (0.68–0.90 *τ*_u_)

Under increasing tensile stress, slip progresses toward the free end, as chemical adhesion abates, and the friction resistance transitions from the static phase to a progressively decreasing dynamic phase [10]. At this stage, the bond resistance, comprising both kinetic friction and interlock, is predominantly governed by the latter. The increase in compression between the steel bars and matrix induces inclined microcracks at the crest of the ribs. The microcracks widen and progressively extend toward the specimen’s surface (see Figure 12). The growth rate of the bonding stress decreases compared to that of the OA phase, while the growth rate of the slip accelerates. The bonding stress‒slip curves begin to deviate from the linear elastic stage OA, and this bonding behavior continues until the tensile stress reaches the ultimate tensile strength of the UHPC at the top of the rib (approximately 0.83 *τ*_u_). At this point, the matrix remains largely intact, and mechanical interlock dominates the bond mechanism. Like elastic phase OA, the unloading process fully closes the rib-crest microcracks [39], leaving negligible residual slip.

(3) Stabilized crack propagation phase BC (0.68–1.0 *τ*_u_)

With continued tensile loading, compression between the steel bar and the surrounding matrix intensifies. The inclined microcracks initiated during the AB stage continue to propagate and widen, while additional inclined microcracks appear. At the free end, the sliding becomes more pronounced. The rate of the bond stress growth decelerates relative to that in the AB phase as the slip rises rapidly. The *τ*-*s* relationship exhibits increasingly nonlinear behavior until reaching its maximum bond stress *τ*_u_ (point C) [10]. The high tensile strength of the UHPC matrix and the confining effect of steel fibers mitigate the crack propagation, enabling the interlock and dynamic friction mechanism to remain operative and thereby achieving a higher bond strength [39]. The average *τ*_u_ between NPR and UHPC specimens reported in this paper is approximately 43 MPa.

(4) Unstable crack propagation phase CD (*s*_u_ − *s*_r_)

Beyond *τ*_u_, continued loading triggers the development of numerous unstable microcracks, accelerating damage accumulation. The matrix ahead of the ribs undergoes severe crushing (see Figure 12), permitting significantly greater slip than prior to *τ*_u_. Consequently, interlocking forces are reduced to the point of ineffectiveness against tensile stresses, leading to a gradual decline in the bond stress. This behavior manifests as pronounced softening of the bond stress–slip curve.

(5) Residual stage DE (>*s*_r_)

As the loading continues, the concrete keys within the ribs are nearly completely sheared off, with ongoing rebar movement reducing them to a powder, thereby nearly abolishing the interlocking mechanism. Steel fibers lose their bridging functionality. The dense matrix filling the spaces between the ribs creates dynamic friction as the rebar is pulled out. Consequently, the primary source of residual bond strength is this residual dynamic friction, which prevents the bond stress from decreasing to zero and maintains a relatively stable bond stress at approximately 0.31*τ*_u_ until the rebar is fully pulled out from the UHPC.

Table 5 summarizes the test results. Among them, (*τ*_cr_, *s*_cr_) corresponds to point B in Figure 12. Here, *P*_u_ is the peak load (kN), and *σ*_sm_ is the rebar’s maximum average stress.

## 5. Discussion

### 5.1. Effect of the Rebar Type

**Figure 13:**
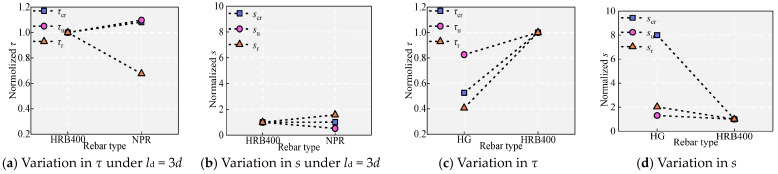
Effect of rebar type: (**a**,**b**) NPR and HRB400 and (**c**,**d**) HRB400 and HG.

Figure 11 and Figure 13 show the effect of rebar type on bond performance. As shown in Figure 11, when *l*_d_ is 6*d*, the HRB400 bar fractures, while the NPR bar yields, and its *σ*_sm_ reaches 892.22 MPa. Table 5 shows that at *l*_d_ = 3*d*, the HRB400 bar yields (*σ*_sm_ = 540.67 MPa), whereas the NPR bar does not yield (*σ*_sm_ = 593.00 MPa). The slope of the rising section of specimen B is greater than that of specimen M during initial loading. This indicates that the initial bond stiffness between NPR and UHPC is greater than that between HRB400 and UHPC. The *τ*_cr_ and *τ*_u_ of specimen B (NPR) are larger than those of specimen M (HRB400). The larger *τ*_u_ is mainly due to two reasons. On the one hand, during the initial loading phase, there is no slip between the UHPC and rebar. Rebar with higher yield strength possesses relatively greater resistance to slippage, thus experiencing a longer initial period of shared loading with UHPC and consequently exhibiting higher bond stress [5]. This is also the reason why the *τ*_cr_ of specimen B is greater than that of specimen M. On the other hand, as the tensile load increases, the HRB400 bar yields and enters a plastic phase, where the rebar elongates and thins [8], which reduces the contact area with the matrix, and the interlocking and friction forces decrease. Conversely, under the same tensile load, the NPR rebar remains in the elastic stage. The contraction due to the Poisson effect has a lesser impact on the bond stress, resulting in greater frictional and interlocking forces [5]. Due to the high yield strength of rebar, the slip caused by the elongation of rebar under strain permeation decreases. Therefore, the *s*_u_ of specimen B is significantly less than that of specimen M. Specifically, the *s*_u_ for specimen B is 0.80 mm, while for specimen M, it is 1.52 mm. After approximately 6 mm, the rate of the descending phase of specimen B is significantly greater than that of specimen M, mainly because after 6 mm, specimens B and M underwent shearing of the interlocking teeth. In specimen M, when the shearing of interlocking teeth occurs, the steel fibers continue to provide tensile bridging within the matrix, leaving a rough shear surface due to numerous fibers remaining within the bond length. Conversely, in specimen B, most fibers are either pulled out or sheared off when the interlocking teeth are sheared [20], resulting in fewer residual fibers within the bond length and a notably smoother shear surface compared to specimen M.

As shown in Figure 11 and Figure 13c,d, the bond–slip characteristics of the HG bars are significantly different from those of the HRB400 bars. Specifically, compared to specimen P (HRB400), the bond stiffness in the rising section of specimen O (HG) is significantly smaller, and the bond stress at different characteristic points is significantly smaller, while the corresponding slip is significantly larger. Compared with specimen P, the *τ*_u_ of specimen O decreased by 17.43%, and *s*_u_ increased by 31.02%. Notably, for specimen P, *s*_u_ also contains the slip generated by the elongation of the rebar since the HRB400 rebar of specimen P has yielded (*σ*_sm_ = 600.69 MPa). In contrast, the HG steel bars in specimen O (*σ*_sm_ = 495.87 MPa) remain in the elastic phase. Therefore, in reality, the *s*_u_ of specimen O is significantly greater than that of specimen P by more than 29.06%. When the slip reaches approximately 20 mm, the bond–slip curve of specimen O does not continue to decrease but instead exhibits a continuous wave-like curve with gradually decreasing amplitudes. The wavy curve is due to the unique spiral groove shape of the HG rebar.

In conclusion, the NPR rebar is superior to the HRB400 rebar, and the HRB400 rebar is superior to the HG rebar in terms of bonding with UHPC.

### 5.2. Effect of Pretension Strain

The effect of the rebar stress‒strain relationship on bond performance depends on a combination of the stress conditions and material properties. Typical medium- and large-scale tests performed on ductile members and sub-assemblages result in significant changes in bond behavior due to rebar yielding [28]. In contrast, most previous bond–slip tests have been set up to ensure that the rebar stays within the elastic range. Only a few studies have examined the bonding of rebar when it is in the inelastic range [28]. In this study, significant reinforcement yielding occurred in specimens D and 2 with anchorage lengths of 5*d* and 6*d*, respectively. Moreover, the characteristic uniform tensile elongation properties of NPR bars occur in the inelastic range, which is significantly different from the local necking that occurs in HRB400 bars in tension. Furthermore, the ultimate tensile strain of the NPR bars is approximately double that of the HRB400 bars.

**Figure 14:**
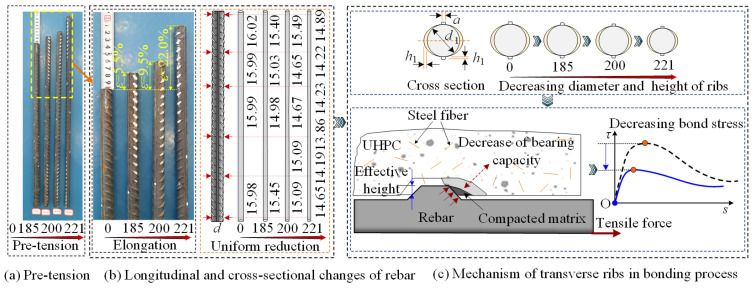
Mechanism of the effect of pretension strain.

To explore the bond behavior after rebar yielding, specimens 11, 12, and 13 were prestressed with pretension forces of 185 kN (corresponding strain of 5.5%), 200 kN (strain of 9.5%), and 221 kN (strain of 22.0%), respectively, before casting. Figure 14a,b show the longitudinal elongation and cross-sectional diameter *d* variation in the NPR rebar under different pretensions. Figure 11 and Figure 14 show the effect of pretension strain on bond performance.

**Figure 15:**
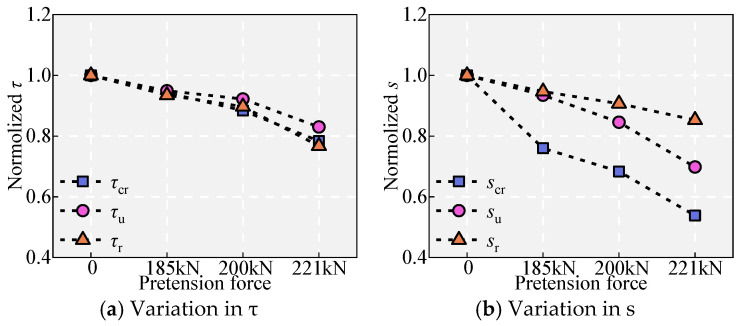
Effect of pretension strain.

As shown in Figure 11 and Figure 15, with increasing pretension, the initial bonding stiffness remains basically consistent, while the bonding stress and slip under different states gradually decrease. Specifically, *τ*_u_ decreased by 5.07%, 7.79%, and 17.01% sequentially. *s*_u_ decreased by 7.00%, 15.88%, and 30.54% sequentially. There is a significant deterioration in the bonding properties of the rebar after yielding. This change is attributed to the inelastic contraction of the ribs, which decreases the mechanical interlock and the interface friction force. The effect of Poisson’s ratio can be visualized from the fact that when the tensile strain is large, the reinforcement yields and “softens”, and Poisson’s ratio *v* significantly exceeds 0.25 in the elastic state. The *v* values of the NPR bar were as high as 0.46, 0.44, and 0.42 at pretension forces of 185 kN, 200 kN, and 221 kN, respectively. Figure 14c illustrates the reduction in the effective rib height due to the uniform elongation of the rebars, resulting in a decrease in bond strength.

### 5.3. Effect of the Rib Height of the NPR Bars

**Figure 16:**
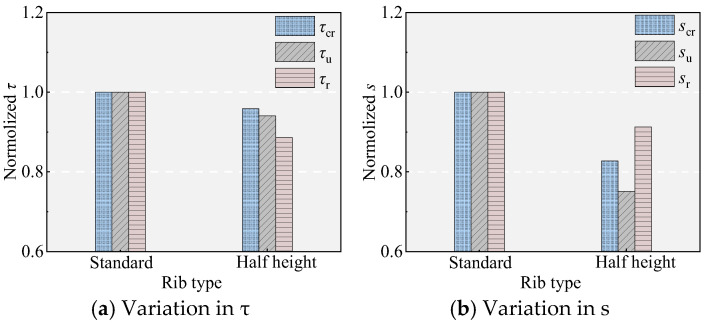
Effect of rib height.

Figure 11 and Figure 16 show the effect of rib height on bond performance. Compared to specimen B, specimen 9 demonstrated the same initial bond stiffness but exhibited decreased bond strength and slip values. Specifically, *τ*_u_ decreased by 5.94%, and *s*_u_ decreased by 24.50%. These changes are attributed to the reduction in rib height, which results in a decrease in the depth of interlocking teeth, leading to susceptibility to shear failure of the interlock teeth.

### 5.4. Effect of the Rib Spacing of the NPR Bars

**Figure 17:**
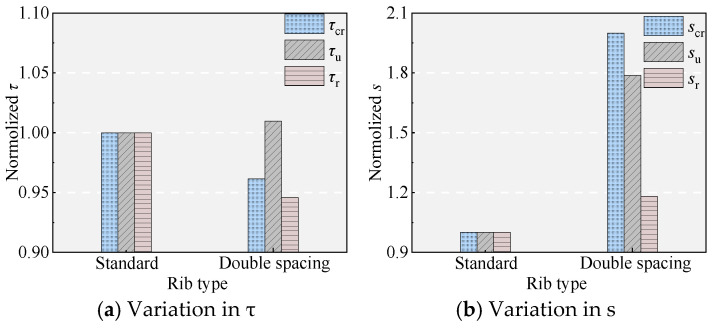
Effect of rib spacing.

Figure 11 and Figure 17 show the effect of rib spacing on bond performance. Compared to specimen B, the increase in the rib spacing in specimen 9 allows more UHPC to fill between the ribs, enhancing the shear area of the UHPC between the ribs. However, this does not improve the bond performance as expected. In contrast, although it has essentially no effect on the bond stress (consistent with the findings of Chang [20]), it significantly reduced the bonding stiffness before reaching *τ*_u_ and increased *s*_u_ (increased by 79.65%), which is unfavorable for bonding performance.

### 5.5. Effect of the Embedment Length l_d_

**Figure 18:**
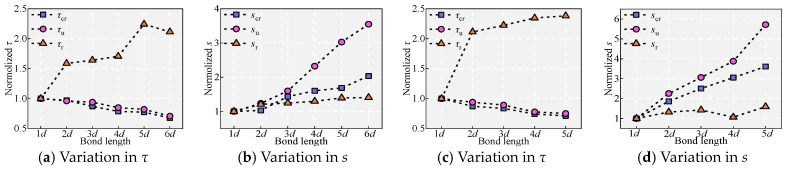
Effect of *l*_d_: (**a**,**b**) under *V*_f_ = 2.2% and (**c**,**d**) under *V*_f_ = 0.5%.

Figure 11 and Figure 18 show the effect of *l*_d_ on the bond performance. *F*_cr_ increases slightly with *l*_d_, but *τ*_cr_ decreases gradually after averaging. This is mainly because the bond stress is transferred from the loaded end to the free end, and a larger *l*_d_ means a longer transfer path for the bond stresses [36]. With increasing *l*_d_, the peak load *P*_u_ increases significantly, and the decreasing phase after *τ*_u_ becomes relatively flat, exhibiting good bond ductility. This is mainly due to the increase in *l*_d_, which allows more ribs to interlock with the UHPC, thereby restricting the decrease in bond stress. As *l*_d_ increases, *s*_u_ gradually increases, while *τ*_u_ gradually decreases, indicating that within the range of *l*_d_ < 6*d*, the weakening effect of uneven stress distribution on bond stress is greater than the strengthening effect of interlocking. Furthermore, integrating the bond equilibrium equation $\tau +\frac{d}{4}\frac{d\sigma_{s}}{dx}=0$ along the anchorage length yields $\tau =\sigma_{s}(0)\frac{d}{4l_{d}}$ (*σ*_s_(0) is the rebar stress at the loading end), which once again demonstrates the inverse relationship between *τ*_u_ and *l*_d_. Both *τ*_r_ and *s*_r_ increase with increasing *l*_d_. This is because, for specimens experiencing splitting–pull-out and pull-out, when the slip is sufficient, the concrete teeth between the ribs are completely sheared or crushed, and the shear keys between the ribs and concrete are almost exhausted, resulting in the basic disappearance of interlocking. In this state, *τ*_r_ is mainly generated by friction. A larger *l*_d_ implies more contact between the ribs and the matrix, thus leading to greater friction. The *σ*_sm_ for specimen C (*l*_d_ = 4*d*, *V*_f_ = 2.2%, *c*/*d =* 2.2) is 713.94 MPa. The *σ*_sm_ for specimen U (*l*_d_ = 5*d*, *V*_f_ = 0.5%, *c*/*d =* 2.2) is 701.06 MPa. This indicates that the critical embedded length is 5*d* for *c*/*d* = 2.2 and *V*_f_ = 0.5% and 4*d* for *c*/*d* = 2.2 and *V*_f_ = 2.2%.

Notably, with increasing *l*_d_, a decreasing pattern in *τ*_u_ has been observed by most researchers [2,10,40,41]. This finding was also observed in Rao’s [40] study on high-strength concrete and ribbed bars, Alkaysi’s [41] research on UHPC with smooth bars and epoxy-coated ribbed bars, and Khaksefidi’s work [10] on UHPC with ordinary (*f*_y_ = 499.8 MPa) and high-strength (*f*_y_ = 600.3 MPa) rebars. Furthermore, Khaksefidi [10], Yoo [2], and Yuan [42] studied NC, high-strength concrete, ultra-high-strength concrete, and UHPC. However, in some investigations, contrary results have been reported [10]. The decrease in *τ*_u_ can be attributed to the increasingly pronounced uneven distribution of bond stress with increasing *l*_d_. Since these studies all adopted average bond stresses, specimens with larger *l*_d_ values exhibited lower *τ*_u_ values. Furthermore, due to the influence of the reinforcement’s Poisson effect, as *l*_d_ increases, the contact area between the rebar and the matrix decreases after yielding, leading to a decrease in *τ*_u_. The increase in *τ*_u_ can be summarized as follows: since the bond stress is significantly influenced by interlocking, a larger *l*_d_ implies that more ribs participate in pull-out, resulting in an increased bond strength *τ*_u_. If the strengthening effect of interlocking outweighs the weakening effects of uneven stress distribution and Poisson’s ratio, then *τ*_u_ and *l*_d_ exhibit a positive correlation; otherwise, they demonstrate a negative correlation.

### 5.6. Effect of Stirrup Spacing s_s_

**Figure 19:**
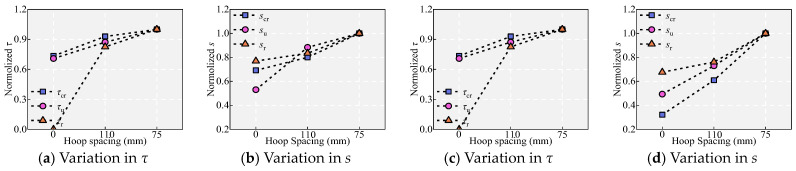
Effect of *s*_s_: (**a**,**b**) under *V*_f_ = 2.2% and (**c**,**d**) under *V*_f_ = 0.5%.

Figure 11 and Figure 19 show the effect of *s*_s_ on the bond performance. The confinement effect of the hoop is mainly reflected in two aspects. On the one hand, the stirrup can effectively limit the cracking of the cover and increase the residual strength of UHPC [43]. On the other hand, after the cover cracks, the radial tensile stress is partially transferred to the stirrups, with the bond stress being jointly supported by the steel fibers and stirrups. Therefore, at a slip of 10 mm (corresponding to the spacing of the ribs), specimens with higher stirrup ratios demonstrated better bond strength retention. Specifically, specimen Z with a *ρ*_ss_ of 1.14% and specimen I with a *ρ*_ss_ of 1.68% maintained strengths *τ*/*τ*_u_ of approximately 0.31 and 0.38, respectively. The final bond stresses in specimens undergoing split–pull-out and pull-out failure are mainly composed of friction. The friction tends to a constant value due to hoop restraint [43], and the friction is positively correlated with the restraint level (i.e., *ρ*_ss_). This can also be seen in the increase in *τ*_r_ in specimens L, B and Z, I. As shown in Figure 11 and Figure 19, the effect of *s*_s_ on bonding performance follows the same pattern at different *V*_f_. With decreasing *s*_s_, i.e., an increase in the hoop restraint, the initial bond stiffness of the specimen remains basically the same, the bond stress and slip in different states both increase gradually, and the bond ductility after *τ*_u_ also increases gradually. At *V*_f_ = 0.5%, *τ*_u_ increased by 17.12% and 31.55%, while *s*_u_ increased by 47.89% and 102.71%. At *V*_f_ = 2.2%, *τ*_u_ increased by 23.11% and 41.16%, while *s*_u_ increased by 66.59% and 88.63%.

### 5.7. Effect of Curing Time

**Figure 20:**
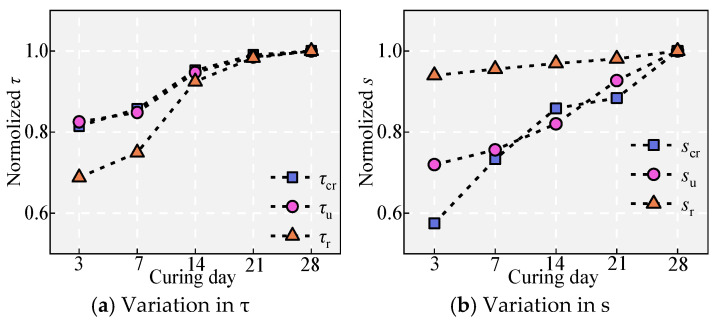
Effect of curing time.

Within 28 days, the strength of the UHPC increases with increasing curing age. Hence, the effect of curing age on bonding performance is essentially the effect of UHPC strength on bonding performance. Figure 11 and Figure 20 show the effect of curing time on bond performance. From these figures, it can be observed that the bond stress and slip under different conditions gradually increase. Among these, *s*_u_ increased by 5.06%, 8.47%, 13.00%, and 7.88%, while *τ*_u_ increased by 2.81%, 11.65%, 4.00%, and 1.51%, respectively. The reasons for the aforementioned changes are the increase in curing age, which enhances the strength of the UHPC, thereby improving chemical adhesion and interlocking. Moreover, the strength of the interlocking teeth of the matrix between the ribs increases, delaying the occurrence of internal splitting cracks in the matrix and thus improving bonding performance [36].

### 5.8. Effects of the Steel Fiber Volume V_f_

**Figure 21:**
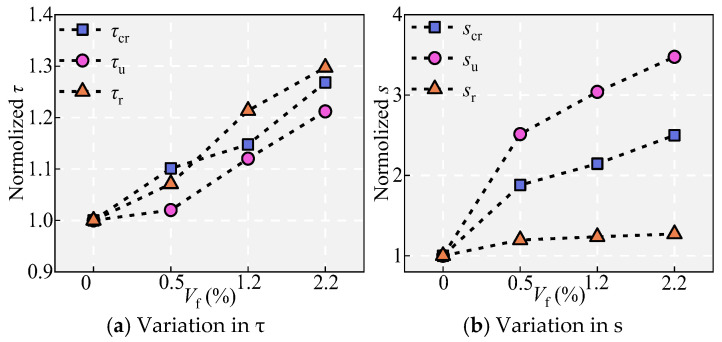
Effect of *V*_f_.

Figure 11 and Figure 21 show the effect of *V*_f_ on the bond performance. As *V*_f_ increases, splitting cracks on the specimen surface become narrower, and the failure modes shift from splitting to pull-out (such as for specimens H, I, and J) to pull-out (such as for specimen B). With increasing *V*_f_, the bond stress and slip in different states both increase gradually, and the bond ductility after *τ*_u_ also increases gradually. Specifically, with increases in *V*_f_ from 0% to 0.5%, 1.2%, and 2.2%, *τ*_u_ increases by 2.01%, 9.81%, and 8.19%, respectively, and *s*_u_ increases by 151.40%, 20.90%, and 14.37%, respectively. The increase in *τ*_u_ and *s*_u_ with increasing *V*_f_ can be explained through the bridging effect of steel fibers [27,39].

### 5.9. Effect of the UHPC Cover Depth c

Splitting cracks appeared on the surface of specimen V, and as *c* increases, the crack width significantly decreases. The appearance of splitting cracks is due to the lack of restraint. As *c* increases, the restraining effect increases, inhibiting the development of internal radial cracks and thereby gradually reducing the width of the splitting cracks [8,36]. The variation in splitting cracks can also be introduced by the theory of thick-walled cylinders.

**Figure 22:**
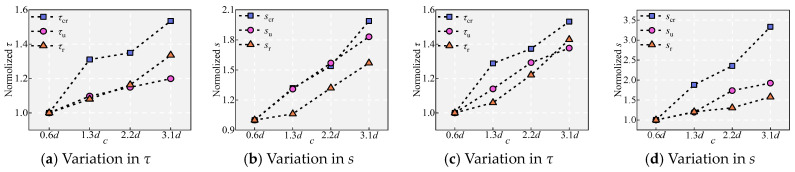
Effect of *c*: (**a**,**b**) under *V*_f_ = 2.2%, and (**c**,**d**) under *V*_f_ = 0.5%.

Figure 11 and Figure 22 show the effect of *c* on the bond performance. The results indicate that with increasing *c*, the bond stress and slip in different states both increase gradually, and the bond ductility after *τ*_u_ also increases gradually. Specifically, with increases in *c* from 10 mm to 20 mm, 35 mm, and 50 mm, *τ*_u_ increases by 13.96%, 13.44%, and 6.53%, respectively, and *s*_u_ increases by 19.44%, 45.39%, and 10.75%, respectively. The growth rate of *τ*_u_ decreases with increasing *c*, indicating that the enhancing effect of increasing *c* on *τ*_u_ gradually diminishes, which is consistent with the findings of Shao et al. [1,11]. The increase in *τ*_u_ with *c* can be explained by using a simplified theoretical derivation. It is assumed that when the specimen develops splitting cracks, the splitting plane extends to the bottom of the rebar, and the UHPC at the splitting plane is still in the strain-hardening stage. From the approximate balance between the UHPC resistance to splitting cracks, represented by 2*f*_t_*cl*_d_, and the bond force π*dl*_d_*τ*_u_, it can be derived that *τ*_u_ = 2*f*_t_*c*/π*d* [24].

**Figure 23:**
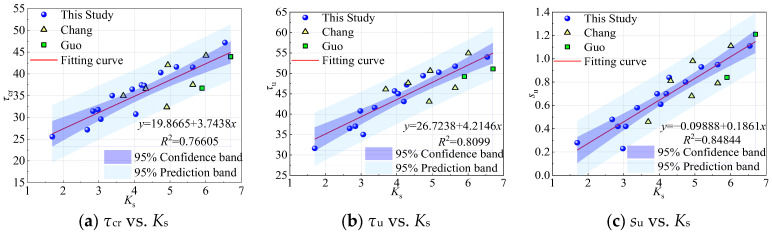
Variation in *τ*_cr_, *τ*_u_, and *s*_u_ with *K*_s_ [15,20].

We further explore the influence of confinement on the bond stress and slip. Figure 23 shows the variations in *τ*_cr_, *τ*_u,_ and *s*_u_ with respect to the confinement parameter *K*_s_ (1.7 < *K*_s_ < 6.7). As shown in Figure 23, there is a positive correlation between the specimen bond stress, slip, and *K*_s_.

## 6. Critical Embedded Length *l*_cd_ and Ultimate Embedded Length *l*_ud_ Models

The anchorage, lap, and extension of reinforcements in concrete should consider the ultimate limit state. The minimum anchorage length required to reach the ultimate limit state is defined as the critical embedded length *l*_cd_. In other words, *l*_cd_ is the bond length at which the loaded end of the rebar has reached the yield state *f*_y_ while the free end remains no slip to prevent the rebar from pulling out before yielding and failing to utilize the strength of the rebar [44]. With the further increase in bond length, the rebar yields and enters the strain-hardening stage, the bond stress of the specimen can still grow slowly until reaching the ultimate tensile strength of the rebar *f*_u_, and the corresponding bond length is the ultimate anchorage length *l*_ud_ [10,44]. The bond length in practical structural design is typically maintained between *l*_cd_ and *l*_ud_, thereby achieving optimal utilization of the total capacity of both reinforcing steel and concrete materials. The lower bound *l*_cd_ guarantees that structural strength requirements are satisfied, while the upper bound *l*_ud_ maximizes economic efficiency, since any bond length exceeding *l*_ud_ offers no further benefit.

Thus, the anchorage length corresponds to the maximum rebar stress *σ*_sm_. By setting *σ*_sm_ equal to *f*_y_ and *f*_u_, respectively, we obtain the values of *l*_cd_ and *l*_ud_. The following section employs regression models and ANN models to establish computational frameworks for *σ*_sm_, aiming to achieve precise prediction of *σ*_sm_.

### 6.1. Multiparameter Regression Model for σ_sm_

Using the values measured in the previous section together with those reported by Guo [15] and Chang [20] (see Table 6), we developed a multiparameter regression model for the prediction of *σ*_sm_. Expressed in the functional form of Equation (2) and presented explicitly in Equation (3), this model incorporates the effects of *l*_d_/*d*, *c*/*d*, *λ*_sf_, *ρ*_ss_, and *f*_cu_. By substituting *σ*ₛₘ = 690 MPa into Equation (3), the *l*_cd_ can be determined for any set of design parameters. Likewise, substituting *σ*ₛₘ = 1139 MPa yields the *l*_ud_ for those same parameters.

(2) $$\frac{\sigma_{\mathrm{sm}}}{\sqrt{f_{\mathrm{cu}}}}=(x_{1}+x_{2}\times \frac{d}{l_{d}})(x_{3}+x_{4}\times \frac{c}{d})(x_{5}+x_{6}\times \frac{l_{f}}{d_{f}}V_{f})(x_{7}+x_{8}\times \frac{A_{\mathrm{ss}}}{bs_{s}})$$

where *x*_1_, *x*_2_, *x*_3_, *x*_4_, *x*_5_, *x*_6_ *x*_7_, and *x*_8_ are the regression parameters calculated by Matlab 2024a Deeplearning Toolbox as follows:

(3) $$\frac{\sigma_{\mathrm{sm}}}{\sqrt{f_{\mathrm{cu}}}}=8.310\times 10^{-2}(1.166-\frac{d}{l_{d}})(7.225+\frac{c}{d})(10.250+\frac{l_{f}}{d_{f}}V_{f})(5.779+\frac{A_{\mathrm{ss}}}{bs_{s}})R^{2}=0.85$$

**Figure 24:**
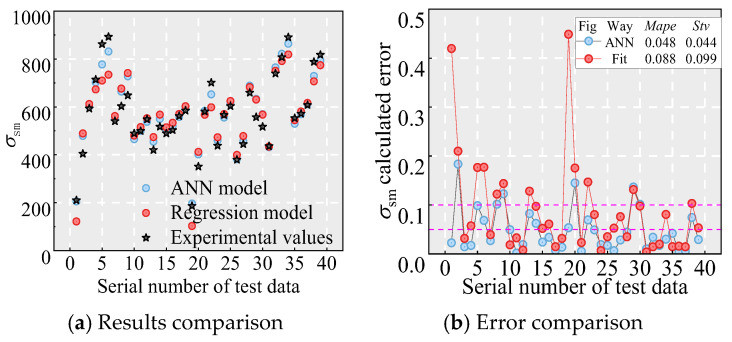
Comparison of the results.

However, as Figure 24 shows, its predictions exhibit large specimen-specific errors. Consequently, the regression model derived from the test data requires further improvement. Previous studies have proven that machine learning algorithms can predict the mechanical indexes of cementitious materials with simplicity and accuracy [45,46,47,48,49,50,51,52,53,54,55]. ANNs can capture complex, nonlinear relationships between design parameters and bond behavior without relying on predefined equations, making them adaptable to diverse datasets [56,57,58,59]. Moreover, ANNs also address the limitations of the regression approach—insufficient accuracy, limited applicability, and high parameter sensitivity—by uncovering latent patterns that regression may miss. Numerous studies report *R*^2^ values above 0.9 when using ANNs to predict bond strength between reinforcement and concrete [56,57,58], and comparisons with other machine learning techniques confirm that ANNs deliver superior predictive performance [56,57,58]. For example, Li et al. [57] assembled a database of 557 bond measurements for rebars in UHPC and evaluated nine machine learning models (four linear, five nonlinear), finding that the ANN achieved the highest accuracy. In light of these existing findings, the ANN can predict mechanical indexes for rebars in UHPC with simplicity and accuracy [56,57,58,59]. Therefore, this paper employs an ANN to predict *σ*_sm_ for NPR rebar in UHPC, thereby establishing a computational model capable of accurately determining *l*_cd_ and *l*_ud_.

### 6.2. ANN Model for σ_sm_

#### 6.2.1. Establishment

The elaborate details of ANN can be found in the work by Yuan, Xiong, Li et al. [42,56,57]. The activation functions of Tanh were adopted in the hidden layers and output layer to introduce nonlinearity and adjust the output appropriately. The parameters were first normalized to accelerate the speed of gradient descent [59]. The loss functions of mean squared error (MSE) and the SGD (Stochastic Gradient Descent) optimizing method are then introduced. After inputting data, the model first undergoes forward propagation, where inputs pass through the network to generate predictions. Then, backpropagation adjusts the weights to minimize the loss by calculating gradients and updating the weights accordingly. This cycle repeats for several epochs [58]. The detailed establishment process of the ANN model can be referred to in references [42,59,60].

##### Database

**Figure 25:**
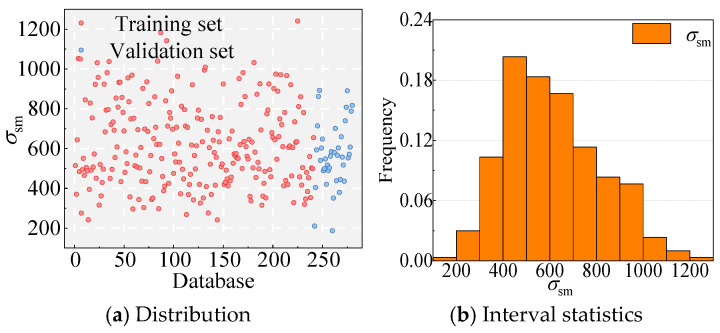
Database.

Given the scarcity of NPR’s bond–slip data with UHPC, limited to this study, Chang’s study [20], and Guo’s study [15], an approach similar to that of Yoon et al. [59] was adopted to satisfy the data volume requirements of the ANN. First, feature engineering is performed, after which Equation (3) is applied with the introduction of noise to mitigate overfitting, enlarging it to 241 training samples [59]. For a full description of the approach, refer to Yoon’s study [59]. Interval statistics for both the validation and training sets are displayed in Figure 25.

##### Input and Output

**Figure 26:**
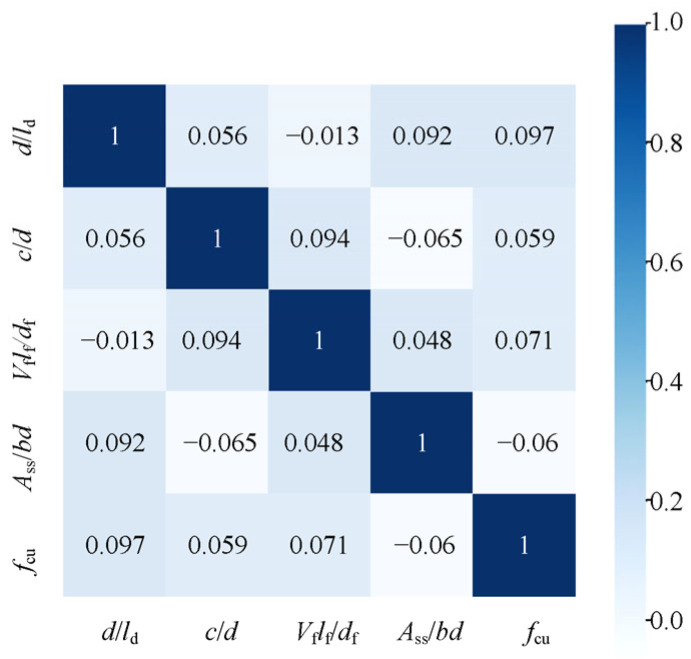
Heatmap of *R*^2^.

The established ANN model utilizes several input variables, including *A*_ss_, *b*, *s*_s_, *l*_d_, *d*, *V*_f_, *l*_f_, *d*_f_, *f*_cu_, *c*, and curing time *t*. The output variable is *σ*_sm_. Pearson correlation analysis was conducted to assess whether input variables require dimensionality reduction [57]. The resulting correlation matrix is depicted in Figure 26, with the relevant formula provided in Equation (4). As indicated in Figure 26, the highest observed correlation coefficient *R*^2^ is 0.097, suggesting that dimensionality reduction is unnecessary.

(4) $$r=\frac{\sum_{i=1}^{n}\left(X_{i}-\bar{X}\right)\left(Y_{i}-\bar{Y}\right)}{\sqrt{\sum_{i=1}^{n}{\left(X_{i}-\bar{X}\right)}^{2}}\sqrt{\sum_{i=1}^{n}{\left(Y_{i}-\bar{Y}\right)}^{2}}}$$

where *X_i_* and *Y_i_* represent the different input parameters, respectively; $\bar{X}$ and $\bar{Y}$ are the mean value of the specific different input parameters.

##### Evaluation Indicators

The accuracy of the ANN model is assessed using the *R*^2^, the mean absolute percentage error (*MAPE*), and the root mean square error (*RMSE*). The calculating formula is as follows:

(5) $$R^{2}=1-\frac{\sum_{i=1}^{n}{\left(X_{\mathrm{calc}}-X_{\mathrm{test}}\right)}^{2}}{\sum_{i=1}^{n}{\left(X\mathrm{test}-{\bar{X}}_{\mathrm{test}}\right)}^{2}}$$

(6) $$MAPE=\frac{100\%}{n}\sum_{i=1}^{n}\left|\frac{X_{\mathrm{calc}}-X_{\mathrm{test}}}{X_{\mathrm{test}}}\right|$$

(7) $$RMSE=\sqrt{\frac{1}{n}\sum_{i=1}^{n}{\left(X_{\mathrm{calc}}-X_{\mathrm{test}}\right)}^{2}}$$

where *X*_test_ represents the test value; ${\bar{X}}_{\mathrm{test}}$ represents the mean of test values; *X*_calc_ represents the calculated values; and ${\bar{X}}_{\mathrm{calc}}$ represents the mean of the calculated values.

##### Modeling

**Figure 27:**
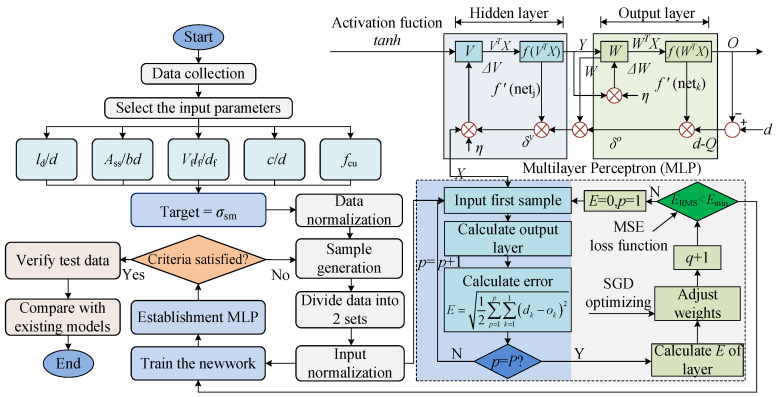
ANN modeling [31].

A two-layer ANN model was developed using the backpropagation (BP) algorithm. The network configuration consists of four hidden nodes, with the *tanh* activation function applied, as specified in Equation (8). The learning rate is set to 0.01. The gradient descent method is employed for optimization to minimize the loss function. The batch training procedure of the BP algorithm is illustrated in Figure 27. Additionally, Figure 27 demonstrates the process of constructing the ANN-based model to determine the *σ*_sm_ for UHPC specimens reinforced with NPR bars.

(8) $$\tan h(x)=\frac{2}{1+e^{-2x}}-1$$

In accordance with the previously outlined criteria, the neural network architecture consists of an input layer with five neuron nodes, an output layer with four neuron nodes, and two hidden layers. Both the input and output values are normalized to the range of −1 to 1, with a noise factor of 0.02, and the dataset comprises 280 samples. Among all ANN models in gird search, the final selected ANN has the highest *R*^2^ and lowest *MSE*.

#### 6.2.2. Verification

**Figure 28:**
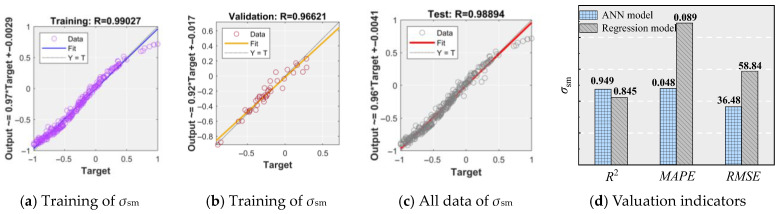
Valuation indicators.

Figure 24 and Figure 28 illustrate the comparison between the established ANN model, including the regression model, and the experimental values, along with their respective error analyses. Figure 28 presents the comparison of evaluation metrics. As shown, the ANN model demonstrates the strong correlation between inputs and targets, closely matching experimental values with minimal error, indicating high predictive accuracy for *σ*_sm_. Compared to the regression model (see Equation (3)), the ANN model shows a significant improvement in performance. The *R*^2^ for the ANN validation set is 0.966, substantially higher than that of the regression model. Moreover, the *MAPE* of the ANN model is only 53.9% of that of the regression model, while the *RMSE* is just 62.0%. These results confirm that the ANN model provides markedly higher prediction accuracy for *σ*_sm_ than the regression model. In summary, the established ANN model is highly effective for predicting *σ*_sm_ and can be used to predict the *l*_cd_ and *l*_ud_ between the novel NPR rebar and UHPC. It should be noted that the model’s reliability depends on the quality and representativeness of its training data; extrapolation beyond the calibrated range, unaccounted variability in material behavior, or data noise may introduce errors if directly applied to the practical project, necessitating cautious validation before engineering deployment.

#### 6.2.3. Relative Importance

**Figure 29:**
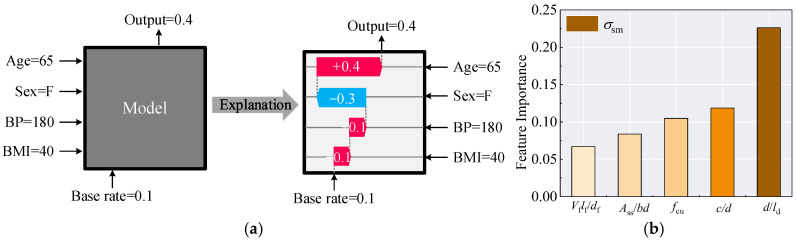
Relative importance. (**a**) Operational principle [44,61]; (**b**) Calculation results.

Feature importance measures the contribution of each input feature to the model’s prediction results, highlighting the relevance of each feature to the target. It helps in understanding both the dataset and the model, supporting the design of structural components. For the ANN, Shapley Additive Explanations (SHAP) theoretical computation is used to assess the influence of input parameters on model outcomes [61], and the operational principle is illustrated schematically in Figure 29a. The SHAP module was imported using Python 3.12, and the results are presented in Figure 29b. As shown in Figure 29b, for *σ*_sm_, the bond length *l*_d_/*d* is identified as the most influential factor, with its importance significantly exceeding that of the other parameters. This finding offers a new perspective for future research on *σ*_sm_.

## 7. Conclusions

In this study, an in-depth investigation of the bond performance between novel NPR bars and UHPC was conducted through eccentric pull-out tests. Based on the test results, a bond characteristic prediction model between NPR bars and UHPC was developed using an ANN, and a three-stage constitutive model was established. The main conclusions are summarized as follows.

(1) Without stirrup confinement, *c*/*d* > 1 (*V*_f_ ≥ 2%) can be considered the minimum cover thickness to prevent splitting failure. With stirrup confinement (*ρ*_ss_ ≥ 1.14), *c*/*d* ≥ 0.63 (*V*_f_ ≥ 0.5%) can be considered the minimum cover thickness to prevent splitting failure.
(2) For UHPC application, the modified constraint parameter *K*_c_ is proposed as the discrimination index to characterize the failure type in this study. *K*_c_ is a combined constraint parameter determined by the stirrups, cover depth, and steel fibers. When *K*_c_ ≤ 4.3, the NPR-UHPC specimens undergo splitting failure. When 4.3 < *K*_c_ ≤ 5.64, the NPR-UHPC specimens undergo splitting–pull-out failure. When *K*_c_ ≥ 5.6, the NPR-UHPC specimens undergo pull-out failure.
(3) In terms of interfacial bonding performance of NPR with UHPC, the NPR bars outperform the HRB400 bars, and the HRB400 bars outperform the HG bars. The unique characteristic of uniform elongation under high tensile strain causes the NPR bars to undergo yield “softening”, which reduces their bonding performance with UHPC, and this reduction is directly proportional to the tensile strain. For the NPR bars, prestrain levels of 5.5%, 9.5%, and 22.0% decrease *τ*_u_ by 5.07%, 7.79%, and 17.01% and *s*_u_ by 7.00%, 15.88%, and 30.54%, respectively. Increasing the rib spacing and reducing the rib height are detrimental to bond performance.
(4) It is discovered that as *l*_d_ increases, *τ*_u_ decreases, while *s*_u_ and *τ*_r_ increase, and better bond ductility is exhibited after the peak point. Decreasing *s*_s_ and increasing the curing age, *V*_f_, and *c* are beneficial for the bond behavior between NPR bars and UHPC, enhancing *τ*_u_ and *s*_u_, and showing better bond ductility after the peak point.
(5) A regression model considering the effects of *l*_d_/*d*, *A*_ss_/*bs*_s_, *V*_f_*l*_f_/*d*_f_, *c*/*d*, and *f*_cu_ is developed to predict the *l*_cd_ and *l*_ud_. Moreover, an ANN model is developed to accurately predict the *l*_cd_ and *l*_ud_ between NPR bars and UHPC. Further, compared to the regression model, the ANN model exhibits a higher *R*^2^ value, lower *MAPE* and *RMSE*, and a broader parameter applicability range.

## Figures and Tables

**Table 1:** Chemical composition and content of rebar (%).

Material	Fe	C	Si	Mn	P	S	Ni	Cr	Cu
NPR	73.59	0.49	0.49	20.2	0.03	<0.01	0.01	3.65	0.02
HRB400	97.78	0.28	0.47	1.54	0.019	0.017	0.005	0.029	0.007

**Table 2:** Mechanical properties.

Type	*d* (mm)	*E*_s_ (MPa)	*f*_y_ (MPa)	*ε*_y_	*f*_u_ (MPa)	*f*_u_/*f*_y_	*ε*_max_	*ε*_u_	Elongation Till Fracture
NPR	16	189	689	5731	1139	1.65	257,173	260,254	28.10
HRB400	16	198	448	2363	583	1.30	136,315	182,590	18.70
HRB400	12	191	433	2348	602	1.38	135,927	193,196	19.80
HG	12.6	196	1386	9047	1471	1.06	38,734	76,379	7.74

Note: the unit of strain is με; *ε*_max_ is the maximum force elongation; and *ε*_y_ and *ε*_u_ are the yield and ultimate strains of the reinforcement, respectively.

**Table 3:** Mixture proportions of UHPC (kg/m^3^) and parameters of steel fibers [31].

Cement	Silica Fume	Quartz Powder	Beads	Quartz Sand	Water	Superplasticizer
797.0	71.0	200.9	31.7	1002.6	198.1	8.2
Diameter (mm)	Length (mm)	Aspect ratio	Young’s modulus (GPa)		Tensile strength (MPa)	Density (kg/m^3^)
0.2	16	80	200		2500	7850

**Table 4:** Test matrix.

No.	Rebar Type	*d*	*l*_d_	*l*_d_/*d*	*c*	*c*/*d*	*V*_f_	*s*_s_	Curing Time	Transverse Rib Shape	Pretension
A	NPR	16	16	1	20	1.3	2.2	75	28	Standard	0
RR	NPR	16	32	2	20	1.3	2.2	75	28	Standard	0
B	NPR	16	48	3	20	1.3	2.2	75	28	Standard	0
C	NPR	16	64	4	20	1.3	2.2	75	28	Standard	0
D	NPR	16	80	5	20	1.3	2.2	75	28	Standard	0
2	NPR	16	96	6	20	1.3	2.2	75	28	Standard	0
E	NPR	16	48	3	10	0.6	2.2	75	28	Standard	0
F	NPR	16	48	3	35	2.2	2.2	75	28	Standard	0
G	NPR	16	48	3	50	3.1	2.2	75	28	Standard	0
H	NPR	16	48	3	20	1.3	0	75	28	Standard	0
I	NPR	16	48	3	20	1.3	0.5	75	28	Standard	0
J	NPR	16	48	3	20	1.3	1.2	75	28	Standard	0
K	NPR	16	48	3	20	1.3	2.2	0	28	Standard	0
L	NPR	16	48	3	20	1.3	2.2	110	28	Standard	0
M	HRB400	16	48	3	20	1.3	2.2	75	28	Standard	0
14	HRB400	16	96	6	20	1.3	2.2	75	28	Standard	0
O	HG	12.6	37.8	3	20	1.6	2.2	75	28	Standard	0
P	HRB400	12	36	3	20	1.7	2.2	75	28	Standard	0
5	NPR	16	48	3	20	1.3	2.2	75	3	Standard	0
6	NPR	16	48	3	20	1.3	2.2	75	7	Standard	0
7	NPR	16	48	3	20	1.3	2.2	75	14	Standard	0
8	NPR	16	48	3	20	1.3	2.2	75	21	Standard	0
9	NPR	16	48	3	20	1.3	2.2	75	28	Reduce rib height to half	0
10	NPR	16	48	3	20	1.3	2.2	75	28	Increase rib spacing to double	0
11	NPR	16	48	3	20	1.3	2.2	75	28	Standard	185
12	NPR	16	48	3	20	1.3	2.2	75	28	Standard	200
13	NPR	16	48	3	20	1.3	2.2	75	28	Standard	221
R	NPR	16	16	1	20	1.3	0.5	75	28	Standard	0
S	NPR	16	32	2	20	1.3	0.5	75	28	Standard	0
T	NPR	16	64	4	20	1.3	0.5	75	28	Standard	0
U	NPR	16	80	5	20	1.3	0.5	75	28	Standard	0
V	NPR	16	48	3	10	0.6	0.5	75	28	Standard	0
W	NPR	16	48	3	35	2.2	0.5	75	28	Standard	0
X	NPR	16	48	3	50	3.1	0.5	75	28	Standard	0
Y	NPR	16	48	3	20	1.3	0.5	0	28	Standard	0
Z	NPR	16	48	3	20	1.3	0.5	110	28	Standard	0

**Table 5:** Test results.

No.	*τ*_cr_	*s*_cr_	*P*_u_	*τ*_u_	*s*_u_	*τ*_r_	*s*_r_	*σ*_sm_	Failure Type
A	46.26	0.08	42.29	52.59	0.50	9.68	9.79	210.34	Pull-out
RR	44.93	0.09	81.32	50.56	0.62	15.37	11.82	404.44	Pull-out
B	40.30	0.12	119.23	49.42	0.80	15.89	12.17	593.00	Pull-out
C	36.32	0.13	143.55	44.62	1.16	16.52	12.69	713.94	Pull-out
D	35.80	0.14	173.30	43.10	1.51	21.72	13.64	861.92	Pull-out
2	31.28	0.17	179.39	37.18	1.77	20.47	13.77	892.22	Pull-out
E	30.75	0.09	108.64	45.03	0.61	14.71	11.46	540.31	Pull-out
F	41.50	0.14	124.84	51.74	0.95	17.12	15.13	620.90	Pull-out
G	47.20	0.18	130.27	53.99	1.11	19.65	17.99	647.86	Pull-out
H	31.77	0.05	98.39	40.78	0.23	12.25	9.57	489.34	Splitting + Pull-out
I	34.99	0.09	100.37	41.60	0.58	13.12	11.45	499.18	Splitting + Pull-out
J	36.46	0.10	110.22	45.68	0.70	14.86	11.83	548.17	Splitting + Pull-out
K	29.58	0.08	84.47	35.01	0.42	0.00	9.36	420.13	Splitting
L	37.44	0.10	103.99	43.10	0.70	13.10	10.15	517.20	Splitting + Pull-out
M	37.28	0.12	108.71	45.06	1.52	23.52	7.74	540.67	Pull-out
14			120.12					597.44	Rebar failure
O	23.02	0.24	61.83	41.33	2.76	10.18	19.86	495.87	Pull-out
P	43.82	0.03	67.94	50.06	2.11	25.09	9.81	600.69	Pull-out
5	32.85	0.07	98.40	40.78	0.57	10.94	11.44	489.40	Pull-out
6	34.53	0.09	101.16	41.93	0.60	11.91	11.63	503.14	Pull-out
7	38.38	0.10	112.95	46.81	0.65	14.69	11.80	561.78	Pull-out
8	39.91	0.11	117.47	48.69	0.74	15.62	11.93	584.25	Pull-out
9	38.64	0.10	112.15	46.48	0.60	14.09	11.11	557.79	Pull-out
10	38.75	0.24	120.38	49.9	1.43	15.03	14.37	598.72	Pull-out
11	38.00	0.09	113.19	46.91	0.74	14.85	11.52	562.96	Pull-out
12	35.63	0.08	109.95	45.57	0.67	14.26	11.04	546.85	Pull-out
13	31.57	0.06	98.96	41.01	0.56	12.19	10.38	492.17	Pull-out
R	41.89	0.04	37.53	46.66	0.19	5.90	8.04	186.65	Splitting + Pull-out
S	36.52	0.07	70.54	43.86	0.42	12.47	10.62	350.86	Splitting + Pull-out
T	31.11	0.11	116.72	36.28	0.73	13.84	8.57	580.54	Splitting + Pull-out
U	29.82	0.13	140.96	35.05	1.08	14.05	12.78	701.06	Splitting + Pull-out
V	27.16	0.05	88.07	36.50	0.48	12.38	9.51	438.02	Splitting + Pull-out
W	37.30	0.11	113.85	47.19	0.84	15.12	12.41	566.26	Pull-out
X	41.60	0.16	121.29	50.27	0.93	17.67	14.99	603.24	Pull-out
Y	25.56	0.03	76.29	31.62	0.28	0.00	7.74	379.46	Splitting
Z	31.43	0.06	89.35	37.03	0.42	11.93	8.72	444.40	Splitting + Pull-out

**Table 6:** Specimen design of Guo’s [15] and Chang’s [20] studies.

From	No.	*l*_d_/*d*	*c*/*d*	*V*_f_ (%)	*l*_f_/*d*_f_	*b* (mm)	*s*_s_ (mm)	Curing Day	*f*_cu_ (MPa)	*f*_t_ (MPa)	*σ*_sm_ (MPa)
[20]	NPR-H-3d	3	4.2	2.5	72.7	150	null	28	153	11.5	659.04
	NPR-L-3d	3	4.2	2	72.7	150	null	28	139	8.3	545.28
	NPR-S-3d	3	4.2	1	72.7	150	null	28	128	7.2	516.96
	NPR-N-3d	3	4.2	0	72.7	150	null	28	86	4.7	355.68
	NPR-H-4d	4	4.2	2.5	72.7	150	null	28	153	11.5	739.52
	NPR-H-5d	5	4.2	2.5	72.7	150	null	28	153	11.5	807.4
	NPR-H-6d	6	4.2	2.5	72.7	150	null	28	153	11.5	890.4
	NPR-H-3d-30	3	1.9	2.5	72.7	150	null	28	153	11.5	552.96
	NPR-H-3d-40	3	2.5	2.5	72.7	150	null	28	153	11.5	571.56
	NPR-H-3d-50	3	3.1	2.5	72.7	150	null	28	153	11.5	607.56
[15]	F1-HP16-4D82	4	5.1	1	80	180	null	28	139	7.7	787.68
	F2-HP16-4D82	4	5.1	2	80	180	null	28	145	9.6	817.28

## Data Availability

The original contributions presented in this study are included in the article. Further inquiries can be directed to the corresponding author.
